# Microfluidics at Fiber Tip for Nanoliter Delivery and Sampling

**DOI:** 10.1002/advs.202004643

**Published:** 2021-03-15

**Authors:** Antoine Barbot, Dominic Wales, Eric Yeatman, Guang‐Zhong Yang

**Affiliations:** ^1^ AS2M Femto‐st Besançon 25000 France; ^2^ Hamlyn Centre, Institute of Global Health Innovation, Imperial College London London SW7 2AZ UK; ^3^ Institute of Medical Robotics Shanghai Jiao Tong University Shanghai 200240 China

**Keywords:** liquid biopsy, microfluidics, two‐photon polymerization

## Abstract

Delivery and sampling nanoliter volumes of liquid can benefit new invasive surgical procedures. However, the dead volume and difficulty in generating constant pressure flow limits the use of small tubes such as capillaries. This work demonstrates sub‐millimeter microfluidic chips assembled directly on the tip of a bundle of two hydrophobic coated 100 µm capillaries to deliver nanoliter droplets in liquid environments. Droplets are created in a specially designed nanopipette and propelled by gas through the capillary to the microfluidic chip where a passive valve mechanism separates liquid from gas, allowing their delivery. By adjusting the driving pressure and microfluidic geometry, both partial and full delivery of 10 nanoliter droplets with 0.4 nanoliter maximum error, as well as sampling from the environment are demonstrated. This system will enable drug delivery and sampling with minimally invasive probes, facilitating continuous liquid biopsy for disease monitoring and in vivo drug screening.

## Graphical abstract

A microfluidic mount on capillaries bundle for precise nanolitre droplet delivery and sampling is presented. A sub‐millimetric microfluidic chip made by two‐photon polymerization is mounted on a dual capillary assembly. The chip has many passive valves to let only liquid flow to the outside. This allows nanoliter precision delivery and sampling of liquid droplets.

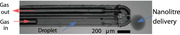

## 1 Introduction

Biopsy is defined as the removal of a small quantity of biological tissue for histopathological examination, and is the gold standard for the diagnosis of many diseases.^[^ ^1^ ^]^ For most cancers, and some infections, it is the only way to characterise the disease precisely, therefore, allowing for precision drug delivery and intervention. However, biopsy can pose risks, cause discomfort and certain side effects to the patient's health.^[^ ^2^, ^3^ ^]^ Therefore, alternative diagnostic techniques, which can complement the gold standard of biopsy for diagnosis of diseases are being pursued.^[^ ^4^, ^5^ ^]^ One such alternative is liquid biopsy. Liquid biopsy is a technique that has garnered substantial attention over the past decade. It is defined as the characterization of tumours by analyzing biomarkers that are circulating in the blood or in other bodily fluids.^[^ ^6^ ^]^ The liquid biopsy technique allows for a more comprehensive sampling of the tumours that may be present and the technique can reduce the need for conducting difficult and risky biopsies from organs such as the brain.^[^ ^7^ ^]^ With the increasing demand for targeted therapy and cell‐based intervention, there is also a growing interest in the development of new microtools that can be used for in situ, in vivo, in vitro applications. Thus, combining the liquid biopsy technique with microtool technologies for targeted therapy and cell‐based intervention is clinically advantageous. With less invasive sub‐millimetric tools at the micron scale for liquid biopsy, certain negative effects could be reduced, while facilitating new interventions and introducing the possibility of long‐term tethered devices or implants for local monitoring of diseases. Indeed, combining sampling with a capacity to deliver small drug liquid volume could facilitate local destruction of a targeted tissue/cells followed by liquid biopsy sampling of the intracellular chemical composition.

However, with size reduction, the biopsy sample volume needs to be reduced, and thus delivering and sampling nanoliter volumes at the tip of sub‐millimeter slender tools are challenging. Indeed, the conventional approach of a simple tube is limited as dead volume (which corresponds to the inner volume of the tube necessary to fill from one end to get liquid at the other end) implies large samples volumes. Moreover, bending perturbation changes the overall volume and reduces delivery and sampling precision.

**Figure 1:**
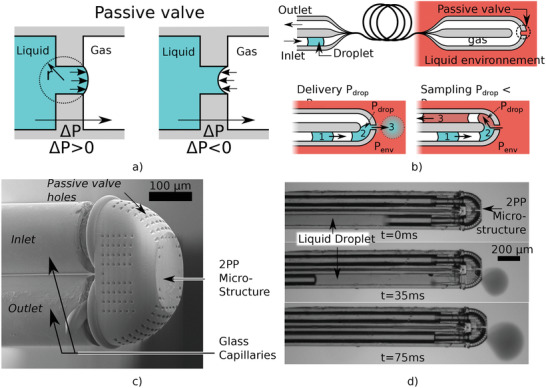
Schematic illustration and microscopic images of the proposed micro‐system for droplet delivery and sampling at a fiber tip. a) Illustrates the concept of a passive valve created at the liquid gas interface. b) Illustrates droplet delivery and sampling mechanisms at the tip of a two capillaries bundle linked together at the tip. A small opening to the environment acts as a passive valve that allows liquid exchange (either out for delivery or in for sampling) only when a droplet is present. c) Shows a Scanning Electron Microscope (SEM) image of the Two‐Photon Polymerization (2PP) structure assembled at the tip of a capillary bundle to provide both connection between the two capillaries and with the outside through several holes acting as passive valve. d) A Video frame showing the transport and delivery of an ink droplet inside the environment.

Following from the digital microfluidic solutions in ref. [8], we propose a two capillary bundle solution for droplet based liquid biopsy. To this end, we designed an original approach using liquid droplets propelled pneumatically. It represents an easy way to deliver droplets, in the order of nanoliters, at the capillary tips using a passive valve mechanism which is widely used in microfluidics.^[^ ^9^ ^]^ Indeed, passive valves use the change of wetting conditions to stop liquid/gas interface movement and therefore withstand a pressure difference as illustrated in **Figure** **1**a.

Figure 1b illustrates how such passive valves enable the delivery and sampling of small liquid droplets using a two‐way channel. The liquid/gas interface on the capillary valve allows the gas to circulate between the two channels without being released in the environment. This gas circulation can then be used to convey a liquid droplet at the tip, which will be either delivered into the liquid environment or will trigger sampling, depending on the droplet pressure difference with the environment.

To create such passive valves, we fabricate a sub‐millimeter microfluidic chip that can be assembled at the tip of the capillary bundle as shown in Figure 1c. The fiber tip structure has mainly been realised by Two‐Photon Polymerization (2PP). Direct printing at the fiber tip has been achieved for microlens arrays,^[^ ^10^ ^]^ structured Surface enhanced Raman scattering (SERs) arrays^[^ ^11^ ^]^ and optical waveguides,^[^ ^12^ ^]^ force sensors,^[^ ^13^ ^]^ or pneumatic actuators as in our previous work.^[^ ^14^ ^]^ In this work, we chose to assemble the structure after printing to simplify the fabrication process while providing a more robust fixation resilient to shock and vibration. Such a fabrication process has been demonstrated for optical resonator^[^ ^15^ ^]^ at the tip of optical fiber. 2PP structures were also demonstrated as the micro nozzle of a microfluidic output.^[^ ^16^ ^]^

Much research in microfluidics has focused on the realization of droplets in two phase flow. For example, use of immiscible liquids, such as aqueous droplets in oil flow, has been proposed to realise droplet based microfluidics for performing small scale chemical reactions.^[^ ^17^ ^]^ In addition, the gas droplet formation in water‐based media^[^ ^18^ ^]^ is also a well‐known mechanism. However, the creation of liquid droplets in a gas medium inside microfluidic channels remains challenging, as the gas cannot prevent the contact between the liquid and the wall. This subsequently leads to a change in the wall wetting condition as the droplet passes in the channel, which causes a non‐continuous pressure change associated with a local energy minimum due to hysteresis of the interface line, rendering the repeatable creation of small liquid droplets in a classical microfluidic T junction difficult.

Therefore, we have also demonstrated in this article a way to produce repeatable 10 nanoliter liquid droplets inside a capillary tube through the use of a hydrophobic coating, ensuring the integrity of the droplet during the propulsion.

The article is organised as follows: We first propose a model to comment on the dynamics of a liquid droplet inside a hydrophobic tube. Secondly, we detail the fabrication of the microfluidic 2PP structure and its assembly with the capillary bundle. We also illustrate the connection of this bundle to a pneumatic circuit in order to propel and create the liquid droplet inside a capillary. Thirdly, we record the volume of multiple droplets and comment on the repeatability of the droplet formation, as well as the droplet speed evolution with the driving pressure. Fourthly, we recorded the droplet volume variation while passing different microfluidic structures attached at the capillary bundle tip for different driving pressure and vacuum values. Finally, we demonstrate how the input and output command can be regulated to realise liquid sampling without having to send a droplet to the microfluidic structure.

## 2 Results

### 2.1 Droplet Modelling in a Hydrophobic Tube

For most microfluidic applications, hydrophilic materials or coatings are chosen. This enables a smooth filling of the chip, preventing gas bubble entrapment, and allows a capillary pumping solution.^[^ ^19^ ^]^ While hydrophilic material simplifies the wetting of the chip, it makes it harder to dry as surfaces tend to remain wet to minimise the overall system energy. This is generally not a problem as fully wet conditions are usually desired in microfluidics

**Figure 2:**
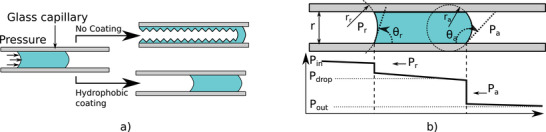
Droplet model in a tube. a) Schematic explaining the droplet size reduction in a hydrophilic tube. b) Model of a droplet propelled in a hydrophobic tube. In the schematic the advancing angle is represented with a value >90° while the receding angle with a value <90°. This model is based on the full wetting and non‐slip condition between the liquid and the wall, which is valid for a static droplet or a slowly moving one.

However, a droplet of liquid propelled in a dry hydrophilic tube will experience size reduction as part of the liquid can remain attached on the tube surface as illustrated in **Figure** **2**a. Therefore, a first requirement to maintain the droplet volume and guarantee that it does not split into different smaller droplets is to use a hydrophobic tube inner surface.

A second requirement to guarantee the droplet integrity is a sufficiently small tube so the capillary forces are dominant compared to gravity. This can be easily checked by using the capillary length defined by :

(1) $$\begin{array}{c} L_{c}=\sqrt{\frac{\gamma }{\rho g}} \end{array}$$

with *γ* the surface tension, *ρ* the liquid density, and *g* the acceleration of gravity. For water, *L*_*c*_ = 2.7 mm so droplets remain easily attached to all the tube section for tube diameters below this value.

Figure 2b shows a schematic of such a droplet with a sketch of the pressure evolution along the tube. One can note the sudden pressure loss at the gas liquid interface which is due to the curvature of the interface. This loss at the interface is known as the Laplace pressure and in a circular tube is expressed as follows:

(2) $$\begin{array}{c} \Delta P=\frac{\gamma }{r^{2}} \end{array}$$

with *r* being the curvature radius of the interface, note *r*_*c*_ and *r*_*a*_ as in Figure 2b. This non‐continuous pressure loss makes important behavior change between having only one or two following droplets in the tube even if the liquid volume is the same. Indeed, when a droplet moves, the difference between the advancing and receding angle at the front and back of the droplet creates a pressure loss. This loss exists even if the droplet is not moving and can therefore prevent motion for small driving pressure.

For two droplets, this loss is double and the minimum pressure for motion is double as well. The pressure loss increases linearly with the droplet numbers thus it is possible for any driving pressure to create enough droplets to block a tube permanently. It is therefore essential to control the created droplet number in order to convey them in a tube in a controllable fashion. In this work we develop methods to limit to a single droplet.

We can therefore derive a model of a moving droplet in a tube with the following assumption :

1. Full wetting of the tube surface and no slip condition at solid/gas and solid/liquid boundaries. However, this assumption doesn't apply to higher droplet speed as shown in Figure 4.
2. Laminar flow. In a tube (circular section) the flow transitions from laminar to turbulent for a Reynolds number (Re) above 2300. In our experiment maximum Re had values less than 15 in both water and air by considering a 100 micron diameter tube and a maximum speed of 1 m s^−1^.
3. Parabolic flow distribution. This is a direct consequence of the laminar flow in a tube. However, this hypothesis is not valid near the gas/liquid interface which imposes constant flow profiles. We decided to make this assumption nonetheless to approximate the drag exerted from the tube wall to the inside fluid.
4. Neglect gravity impact. The droplet volumes were around 10 pL, the weight was therefore equivalent to the force generated by 0.1 mbar pressure on one of the droplet sides. As our pressure control precision is 1 mbar, we decided to neglect gravity impact.
5. Neglect Inertia. As no acceleration regime was noticeable experimentally

The droplet movement is controlled by the pressure at each end of the tube, denoted as *P*_*in*_ and *P*_*out*_ in Figure 2b. This driving pressure applies a force on the droplet, which can be expressed as follows:

(3) $$\begin{array}{c} F_{p}=\pi r^{2}\left(P_{r}-P_{a}\right) \end{array}$$

with *r* the capillary diameter, *P*_*r*_ and *P*_*a*_ the pressure, respectively, at the back and front of the droplet. This can be linked to the driving pressure Δ_*P*_ = *P*_*in*_ − *P*_*out*_ by taking account of the pressure loss due to the friction between the tube and the gas. The friction force from the fluid on a tube section is given by the expression :

(4) $$\begin{array}{c} dF=\mu 2\pi r\frac{\partial v(r)}{\partial n} \end{array}$$

with *μ* the fluid viscosity, *r* is radius, *v* the speed profile, *n* an axis defined by the normal to the surface, and *v* the function that dictates the parabolic flow speed with the distance to the tube center such as *v*(*r*′) = 2*v*_*d*_(1 − *r*′^2^/*r* ^2^), with *v*_*d*_ being the droplet speed. This expression simplifies to

(5) $$\begin{array}{c} dF=-8\mu v_{d} \end{array}$$

Therefore the force on the droplet can be corrected from the pressure loss of the gas :

(6) $$\begin{array}{c} F_{p}=-\pi r^{2}\Delta P-8\mu_{gas}Lv_{d} \end{array}$$

with *L* being the overall tube length.

The same derivation can be done inside the droplet (for which we also assume a parabolic shape), to obtain the friction from the capillary to the droplet, *F*_*d*_ :

(7) $$\begin{array}{c} F_{d}=\pi r^{2}\Delta P-8\mu_{gas}lv_{d} \end{array}$$

With l being the length of the droplet.

The forces induced by both triple contact lines (tube wall, liquid, gas) can be expressed by^[^ ^20^ ^]^:

(8) $$\begin{array}{c} F_{s}=2\gamma r\pi \left(\cos\theta_{r}-\cos\theta_{a}\right) \end{array}$$

with *γ* being the surface tension of the liquid gas interface, *θ*_*a*_ and *θ*_*r*_ respectively the advancing and receding contact angle.

As we neglect inertia (and thus the transition) regime and by considering the drag force from the wall on the droplet, a force balance gives :

(9) $$\begin{array}{cc} & F_{s}+F_{p}+F_{d}=0 \end{array}$$

(10) $$\begin{array}{cc} & \Delta Pr^{2}=2nr\gamma \left(\cos\theta_{a}-\cos\theta_{r}\right)+8v_{d}\left(L\mu_{gas}+l\mu_{liquid}\right) \end{array}$$

with *n* being the number of droplets.

### 2.2 Experimental Setup

The first necessary step was to make the 100 µm capillary hydrophobic. To do so, we utilised an organosilane based treatment, whereby *n*‐octadecyltrichlorosilane was used to form a hydrophobic self‐assembled monolayer (SAM) on the glass surface. This treatment procedure was adapted from a hydrophobic SAM static surface process,^[^ ^21^ ^]^ wherein this work we continuously pumped the organosilane coating solution through the 100 µm glass capillaries (TSP100170 CM‐scientific). The identical treatment made on a flat glass surface exhibited an advancing contact angle of (119.7 ± 0.9)° and a receding contact angle of (96.9 ± 3.9)°, with a statistically significant difference (*p* < 0.01) determined. The resulting contact angle hysteresis is (22.8 ± 4.0)°. These measurements were made on a custom‐made contact angle measurement system following a specific protocol for hydrophobic surface.^[^ ^22^ ^]^ Pictures of the drops from which the contact angles were measured, along with a full description of the measurement procedure, are given in Figure S1, Supporting Information.

The coated capillaries were then assembled to form the delivery/sampling fiber. The overall set‐up, as well as the pneumatic connections, are presented in Figure 2a. A first section of capillary was fitted in a tube with a T junction, allowing the tip to be connected to both a syringe pump and a pressure controller. The syringe pump was used to inject the droplet fluid and was controlled with a manual screw. The pressure controller (Dolomite‐Fluika) set the input gas pressure of the system (*P*_*in*_) and was controlled by a computer. The 2PP structure was fitted inside the capillary. This structure reduced the diameter of the capillary from 100 to 50 µm, therefore providing a passive valve to stop the liquid/gas front and allowed a repeatable droplet volume creation. This structure was printed on a glass substrate, then picked up from the plate with the capillary and pushed inside the tube at approximately 1.5 mm with a 70 µm tungsten filament.

As the depth at which the structure is pushed controls the volume of the droplets, different liquid volume for the micropipette can be selected during the fabrication. The precision at which the valve is placed determines the absolute precision of the pipette. In the reported micropipette the position could be adjusted with one micrometer precision thus with a 0.1 nL accuracy. We call this assembly the nanopipette as it produces 10 nL droplets.

By combining the control of the syringe and the pressure, droplets could be formed in a repeatable way in the tube as shown in 2b) and in Video 2. The protocol was controlled by visual monitoring and was the following:

1. The pressure controller was set to 0 (corresponding to atmospheric pressure). The syringe advanced the liquid front just to the front of the capillary opening.
2. The syringe advanced the liquid front so the capillary filled with liquid. As the 2PP passive valve was not hydrophobic (contact angle around 65°^[^ ^14^ ^]^) it remained wet from previous droplets and the liquid overfilled the tube.
3. The syringe retracted the liquid front. As the capillary was hydrophobic the liquid was removed more quickly than in the containing tube until being stopped by the 2PP passive valve. The main difficulty for this was that the passive valve worked better in stopping a gas front as it is hydrophilic and remains wet.
4. The liquid/gas front in the main tube detached from the capillary opening leaving liquid filled in the capillary before the passive valve, thus forming a droplet. The gas pressure was then raised to open the passive valve and propelled the droplet in the capillary.

A significant challenge was to guarantee that the droplet did not split in two while leaving the nanopipette, as the presence of a second droplet greatly impacts the speed as mentioned in Equation (10) and measured in Figure S2, Supporting Information. Indeed, the passive valve was hydrophilic and remained wet after the passage of the droplet. When gas passes through this wet valve, instability triggering the formation of a small second droplet from the liquid on the surface is possible.^[^ ^23^ ^]^ The speed of the gas flow as well as the amount of remaining liquid on the surface increases the risk of such second droplet occurrence. To overcome this problem the structure of the passive valve was optimised to prevent a remaining liquid pocket. A computer model view of the passive valve is presented in Figure S3, Supporting Information.

At the other end, we assembled the glass tube with a similar hydrophobic capillary to form a two capillary bundle with the end aligned on the same plane. This bundle was fixed using a cyanoacrylate‐based glue. The capillary of the created bundle connected to the nanopipette is the input capillary whereas the other one is the output capillary.

The 2PP microfluidic structure was then printed on glass and assembled directly on the capillary bundle under a microscope. The alignment was achieved using a manual stage (Thorlab‐ Pt3 stages). More details on this process are available in Figure S4, Supporting Information. The microfluidic structure was therefore connected to an input and output capillary.

**Figure 3:**
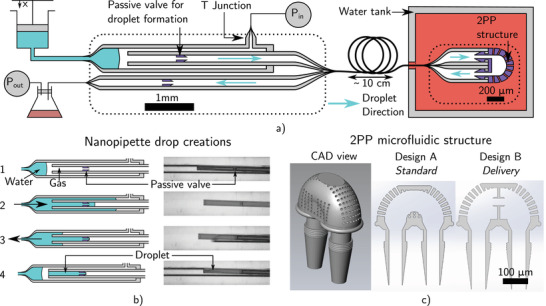
Schematic of the delivery sampling probe. a) Shows a schematic of the overall probe. Control of *x*, *P*_*in*_ *and P*_*out*_ allowed the creation and propulsion of the droplet to the 2PP microfluidic structure and back. b) Schematic and experimental pictures explaining the steps to create a droplet with the nanopipette. c) CAD and cut view of the 2PP microfluidic structure assembled at the capillary bundle tip. Holes between the outside and the environment act as a passive valve, preventing gas to leak into the environment while allowing droplet fluid to pass through.

Two designs of the 2PP microfluidic structure were investigated for this article. The first one (design A on **Figure** **3**c) ) connected the two capillaries of the bundle with no important change on the section. 215 holes with a diameter of 8 µm linked the inner part of the microfluidic chip to the outside environment. These holes act as passive valves and therefore prevent the gas inside the capillary to be released into the environment. It was only when a liquid droplet entered the microfluidic structure that the passive valve opened and that exchanges with the environment were possible. For droplet pressure higher than the environment, the liquid flows from inside the microfluidic structure to the outside allowing the delivery of the droplet. For lower droplet pressure than the environment, the liquid flows from the environment to the inside of the microfluidic structure, allowing liquid sampling to be mixed with the droplet and conveyed back through the output capillary of the bundle. These two behaviors are illustrated in Figure 1b.

The second microfluidic structure (design B on Figure 3c), was designed with a 20 µm diameter passive valve on the main channel. This valve was designed to obtain a more binary behaviour of the microfluidic structure where either all the droplet could be delivered or a continuous sampling could be triggered.

The output capillary was connected to a vacuum generator (*P*_*out*_) in order to get a negative pressure (compared to the environment) contribution to the droplet propulsion. This allowed for control of the droplet pressure difference with the environment while maintaining a constant driving pressure Δ*P* = *P*_*in*_ − *P*_*out*_ as stated in Equation (10). The output capillary end was placed nearby the nanopipette, allowing an easy characterization of the overall system by measuring the input and output droplet from a single microscope view. This end was also equipped with a passive valve to stop the output droplet and simplify its measurement. A picture of the setup is available in Figure S5, Supporting Information.

### 2.3 Measurement of Droplet Speed and Volume Consistency

To characterize the droplet volume and speed, a minimal set‐up was used with only one input capillary that was not connected to the microfluidic structure open in air. Therefore, the droplet was only pushed by the pressure pump and Δ*P* = *P*_*in*_ − *P*_*lab*_ with *P*_*lab*_ the atmospheric pressure during the experiment. The liquid of the droplet was deionised water.

**Figure 4:**
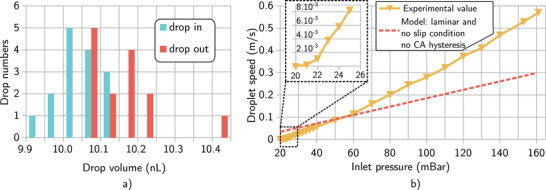
Nanopipette and droplet speed characterization. a) Shows the volume distribution of 15 droplets at their creation in the nanopipette (droplet in) and after travelling 285 mm in the capillary (droplet out) b) droplet speed evolution with driving pressure in a 285 mm capillary. The dashed line represents the maximum allowed speed of the droplet considering full‐wetting and no slip condition on the capillary. Full‐wetting inducing no slip condition is therefore not valid for speed above 0.1 mm s^−1^.

**Figure** **4**a shows the repeatability of the droplet formation and integrity after travelling 285 mm in the capillary. 15 droplets were consecutively created and sent through the tube. After each droplet, a 100 mbar pressure was applied to make sure no residual droplet remains in the capillary. The droplets were measured just after passing the passive valve of the nanopipette (droplet in) and right before exiting the same capillary (droplet out) with a camera mounted on a microscope. An additional passive valve was added near the exit to stop the droplet and simplify its measurement. A record of the characterization of one droplet is available in video 3.

The average volume of the droplet created with the nanopipette was 10.05 nL with a standard deviation of 0.056 nL. The volume difference between the largest and smallest droplet was below 0.2 nL and therefore the nanopipette repeatability error was estimated to be of 2%. On average the volume of the exiting droplet is larger with an average of 10.16 nL with a standard deviation of 0.090 nL.

The speed evolution with the driving pressure was performed in two different ways and is presented in Figure 4b. For driving pressure above 40 mbar, the pressure was raised while a droplet was prepared in the nanopipette. The droplet was therefore released and its speed measured at the end of the capillary to let time for the pressure to reach the desired value. The measurement was performed with a high‐speed camera at 500 frames per second (fps)(340m‐usb) mounted on the microscope. An example measurement is available in Video 3. For pressures below 40 mbar, a droplet was created in the nanopipette and the pressure was briefly raised so the droplet could pass the passive valve and stop inside the capillary. A pulse of the desired pressure was then applied and the speed was measured with the camera set at 199 fps. The same droplet was used to provide all the measurements below 40 mbar whereas one droplet for each point was necessary for the measurements above 40 mbar.

The speed evolution follows a linear trend after a non‐linear regime at speed below 2 × 10^−3^ m s^−1^. Below this value, it is clear that the droplet advances in a jerky fashion by switching between periods of sudden movement and periods where no movement was noticeable. Due to the difference between advancing and receding contact angle, the value for the droplet to start moving is 20 mbar. This value is a bit above the minimum value of 17.1 ± 1.2 mbar which can be deduced from the contact angle measurement on a flat surface and using Equation (10) with *v*_*d*_ = 0.

For comparison, the theoretical speed of the droplet by assuming the above listed hypothesis and no contact angle hysteresis is traced with dash lines. This line is linear and it is directly derived from Equation (10) assuming cos *θ*_*a*_ − cos *θ*_*r*_ = 0 which gives:

(11) $$\begin{array}{c} v_{d}=\Delta P\frac{r^{2}}{8(L\mu_{air}+l\mu_{water})} \end{array}$$

The measured minimum pressure difference to start moving the droplet is 1.17 times higher than the one predicted using the contact angle measurement on a flat surface and Equation (10). This difference may be caused by the difference of surface homogeneity on the tube leading to a larger contact angle hysteresis (cos *θ*_*a*_ − cos *θ*_*r*_) and thus a larger critical pressure for the droplet to move. Indeed, the main reason behind changing the static SAM procedure from the literature^[^ ^21^ ^]^ to a flowing procedure, coupled with substantial solvent rinsing steps at the end of the coating procedure, was to limit the formation of inhomogeneous areas of significant polycondensation causing the organosilane to form organically modified silicate particles, which could restrict or even block the flow through the capillary. However, despite the adapted SAM coating procedure, it should be expected that the roughness and thickness of the SAM coating along the length of the lumen of the capillary may still display some inhomogeneity and this may be increasing the contact angle hysteresis.

### 2.4 Delivery and Sampling Regime Characterization

The two different microfluidic structures (A and B) were mounted successively on a two capillary bundle with a nanopipette producing a 13 pL droplet in the input capillary. The driving pressure Δ*P* = *P*_*in*_ − *P*_*out*_ was set to a constant value of 85 mbar. Variation of droplet volume between the nanopipette and the capillary output were measured. This value indicated the amount of liquid transferred between the microfluidic structure and the environment. Positive value corresponded to flow from the environment to the droplet (sampling behavior) and negative to flow from the droplet to the environment (delivery).

**Figure 5:**
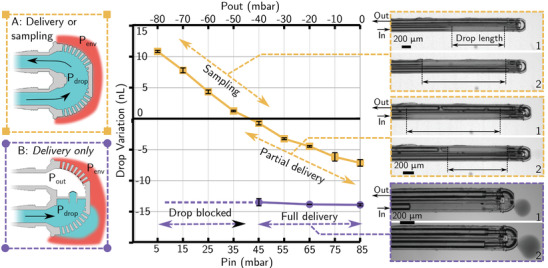
Droplet volume evolution depending on the inlet (*P*_*in*_) and outlet (*P*_*out*_) pressure on the capillary bundle for microfluidic structure A and B set in liquid environment. The difference *P*_*in*_ − *P*_*out*_ was constant and set to 85 mbar to allow constant droplet speed. For high *P*_*in*_ the droplet pressure was higher than the environment and part of the droplet in design A and all of it in design B, is delivered. For low *P*_*in*_ the droplet pressure was lower than the environment and the droplet is sampled. Experimental pictures show the delivery and sampling for design A and correspond respectively to video 4 and 5. A picture of the deliver droplet with design B and ink as liquid is also shown and corresponds to Video 1.

**Figure** **5** shows this variation for different *P*_*in*_ and *P*_*out*_ pairs set to generate an 85 mbar driving pressure. To ease the notation, we consider that 0 mbar pressure corresponds to the environment pressure and that negative pressure corresponds to pressure below this reference. In design A, for pressure less than the pair *P*_*in*_ = 41 mbar, *P*_*out*_ = −44 mbar, pressure inside the droplet is lower than 0 mbar and the liquid from the environment flowed in the droplet as it passed in the microfluidic structure. This corresponds to the sampling regime because part of the environment is captured in the droplet and conveyed back to the capillary exit.

For pressures above this value, the droplet pressure was higher than the environment and a part of the droplet was released, this corresponds to the delivery regime. For both delivery and sampling, Figure 5 shows pictures of the droplet before and after it passed the microfluidic structure. These pictures are extracted from Videos 4 and 5. As only partial delivery and relatively low sampling volume were possible with design A, we proposed design B which had a similar dimension, but an additional passive valve inside the structure made by a 20 µm hole connection. This valve stopped the droplet inside the microfluidic part and allowed its full delivery on all the delivery region. Partial delivery was still possible for values below *P*_*in*_ = 44 mbar, *P*_*out*_ = −41 mbar. However, the end of the droplet was blocked at the microfluidic structure entrance as the diameter of the section was reduced. For easy visualization picture of the experiment and Video 1 are performed with black ink whereas deionised water was used for the measurement.

**Figure 6:**
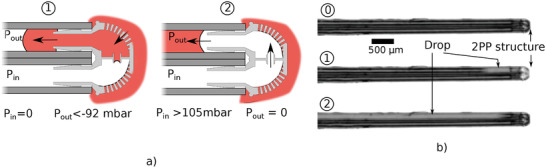
Schematic and experimental demonstration of the sampling with microfluidic design B by only varying *P*_*in*_ and *P*_*out*_. For low *P*_*out*_ value, the environment liquid broke the surface tension seal. Then *P*_*in*_ was increased to propel the sample droplet out of the capillary.

By varying the pressure with time, design B can also sample liquid without the use of a droplet to activate the passive valve. For this *P*_*in*_ was set to 0 mbar while *P*_*out*_ was decreased until the pressure difference was enough for the environment liquid to force through the passive valve and fill the microfluidic part (measured to −92 ± 1 mbar on three trials). A large sampling droplet formed in the output capillary until *P*_*out*_ was set back to zero. Then *P*_*in*_ was raised until the inner passive valve break (measured to 105 ± 1 mbar on three trials) and the droplet on the output capillary could be conveyed to the capillary exit. These steps are illustrated along experimental pictures on **Figure** **6**.

## 3 Discussion

### 3.1 Droplet Volume and Speed

A first interesting result is the increase of the droplet volume as it was propelled inside a single capillary as shown on Figure 3a. This increase may be due to a measurement error caused by different contact angle properties on the tube in the nanopipette and just before the exit. However, one droplet is significantly larger than all the input ones and could be explained by some liquid collection during its travel. Indeed, condensation in the capillary could have occurred on hydrophobic defects while flushing the capillary at 100 mbar between droplet experiments. The condensation droplet should have been small enough not to seal the capillary section and were therefore collected by the following droplet.

The speed value for pressure between 20 and 23 mbar did not follow the clear trend that seems to emerge for higher pressure values. Indeed, as the speed increases, the mechanism of wetting changes from a thermally activated regime (where new surface is wet by thermal agitation of the water molecule ) to a hydrodynamic regime.^[^ ^24^ ^]^ This transition is quantified by the capillary number (*C*_*a*_) which define as :

(12) $$\begin{array}{c} C_{a}=\frac{v\mu }{\gamma } \end{array}$$

with *v* the droplet speed. At 22 mbar the droplet speed is around 1 mms^−1^ which corresponds to *C*_*a*_ = 1.2 × 10^−4^. This corresponds to values in the literature where the transition is known to happen for *C*_*a*_ between 10^−4^ and 10^−5^.^[^ ^24^ ^]^ Measurements specific to hydrophobic surface recorded the stabilisation of the contact angle hysteresis for Ca value above 3 × 10^−4^.^[^ ^25^ ^]^

For higher speed values, a clear trend was observed. However, the speed increase with pressure is roughly two times higher than the one explained in the model describes by Equation (11). While this model made many assumptions, neglecting contact angle hysteresis cannot alone explain this difference as it could only result in reducing the theoretical curve even more. Indeed, the speed curve shown by the dashed line obtained with this model represents the maximum speed possible with the wetting and non slip condition. Therefore, our main assumption is that the friction on the droplet is reduced due to slipping condition between the surface and the liquid. Such slipping phenomenon has been reported on hydrophobic capillaries^[^ ^26^, ^27^ ^]^; however, for a continuous flow and with smaller impact on the flow rate (5% differences reported in the literature). The slipping of the droplet could be also explained by gas entrapment between the droplet and the surface^[^ ^28^ ^]^ that would occur at relatively small speed because of the hydrophobic surface and remain stable due to the small dimension of the tube which increases such stability.^[^ ^29^ ^]^

Full levitating short liquid short droplets (with a maximum length of 5 time the diameter) have also been reported on hydrophillic capillary^[^ ^30^ ^]^ for important capillary number (*C*_*a*_ ≈ 1). In this case, a thin air layer forms between the droplet and the capillary This reduces drastically the droplet friction on the surface and lead to homogenous liquid speed inside the droplet. A model (detailed on Section S6, Supporting Information) based on the work of Favreau et al.^[^ ^30^ ^]^ exhibits a droplet speed that is one order of magnitude higher than our experimental one. Therefore, even if this model is rather simple, it seems unlikely that our droplet is fully detached from the capillary wall. However, partial levitation may happen as instability triggered by irregularities in the hydrophobic coating may produce local rewetting of the droplet on the surface. Such levitating behavior could limit the contamination with the wall as well as reducing drastically the surface friction leading to greater droplet speed. Further relatively simple experiments with higher frame rate would therefore be required to characterize more precisely the behavior of long droplet in hydrophobic capillary.

### 3.2 Sampling and Delivery Regime

The pressure inside the droplet is the key factor to explain the sampling or delivery behaviour. This pressure could be calculated by measuring either the advancing contact angle cos *θ*_*a*_ or the receding one cos *θ*_*r*_ using the Laplace pressure as shown in Equation (2). Unfortunately, these angles need a precise imaging of the moving droplet requiring at least 3000 fps imaging speed for a 0.1 ms^−1^ (we were limited to 500 fps in this study). Moreover, a precise measurement of the dynamic contact angle is difficult and requires a specific experimental design with a moving solid in order to get a static image of the triple line^[^ ^25^, ^31^ ^]^ The droplet speed could be linked to cos *θ*_*a*_ − cos *θ*_*r*_ by Equation (10) which only gives information on the contact angle hysteresis.

Nonetheless, the characterization with varying *P*_*in*_ and *P*_*out*_ pair shown in Figure 5 indicated that the transition between the sampling and delivery regime is well explained by assuming the droplet pressure *P*_*drop*_ = (*P*_*in*_ + *P*_*out*_)/2. Indeed, this transition happens when the average is close to zero and therefore when the pressure in the droplet is the same as the environment. However, even if this approximation is sufficient to predict overall interaction of the microfluidic structure with the environment for one droplet, a closer look at the high‐speed Video 5 shows that the droplet slows and stops in the microfluidic. This speed variation must have an effect on the pressure loss as well as the contact angle and therefore the actual *P*_*drop*_ may be varying during its passage in the microfluidic chip impacting as well on the delivery/sampling flow rate with time.

Design B, with an embedded passive valve was designed to exploit this phenomenon. A passive valve forced the droplet to stay in the microfluidic structure until it is fully delivered. This design is therefore closer to the application as it allows for the delivery of the same volume over a wide pressure range which would be the case for an in‐vivo environment where the pressure is not expected to be constant.

The sampling process illustrated in Figure 6 did not require to send a droplet to trigger the passive valve with the environment It is also a solution to extract larger volume, still without any dead volume, potentially closer to applications of liquid biopsy. For now, the principal limitation is the control of the droplet volume in such a configuration which depends on the negative pressure timing and can be difficult to control.

Additional passive valve integration inspired from the nanopipette design and integrated in the microfluidic structure or the capillary would allow repeatable sampled volume with an automatic pressure cycle. This would render the liquid biopsy fully automatic and more repeatable for a given value set during the microfluidic fabrication. Moreover, the associated control mechanism would only require sequential command of the pump and no precise timing on the applied pressure would be required

## 4 Conclusion

In this article, we first presented a method to create a nanoliter droplet in a hydrophobic capillary. This method allowed the repeatable creation of 10.05 nL liquid droplets with only a 2% repeatability error on the volume. Secondly, we studied the gas propulsion of these droplets in the hydrophobic capillary, demonstrating that speeds up to 0.6 ms^−1^ were possible. We also demonstrated that the relation between the driving pressure and the speed could be modelled as a first approximation by a linear curve for speeds between 5 × 10^−3^ m s^−1^ and 0.6 ms^−1^.

Finally, we proposed a sub‐millimeter slender probe integrating a microfluidic structure at the tip capable of both delivering this nanoliter droplet in a liquid environment as well as sampling a droplet from the environment. This method benefits from the advantage of droplet based microfluidics, such as the absence of dead volume limitations on the precision, allowing for the manipulation of small samples in the nanoliter range.

These results stand as a proof of concept of a potential minimally invasive drug delivery and sampling probe. Such minimally invasive tools could reduce the side effects associated with biopsy. Moreover, the small impact on the targeted organ due to the nanoliter sampling would allow biopsy to be repeated in time. Such time resolved biopsy would enable both in vivo study of organs under different stresses and the monitoring of the progress of diseases such as cancer using an implantable system relying on the small footprint of the probe.

However, many challenges need to be addressed to reach this goal. To this end, our future work will focus on extending the proof of concept to biological challenges such as the use of biocompatible material and surface treatment to guarantee omniphobic contact with biological fluids.^[^ ^32^ ^]^ Automation of the process is also a key challenge to guarantee the repeatability of the delivery and sampling for practical applications. For this, future work should focus on the development of a syringe pump to fill the micropipette feedback with visual tracking of the liquid front.

Investigation on the passive valve design could also allow for adjustment on demand of the micropipette droplet size. Indeed, more complex designs such as succession of passive valve (for a discrete volume selection) or reducing section (for a continuous volume selection with the pressure) could be made. Different microfluidic designs at the tip of the structure will also be investigated in order to control more precisely the sampled volume and to develop cleaning procedures using flush fluid droplet to prevent or limit bio‐fouling or obstruction of the capillary valve holes.

Finally, this work showed that the dynamics of long droplets in hydrophobic channels still need to be resolved. Therefore, further experiments with higher frame rate should be performed to characterize the contact between the moving droplet and the hydrophobic capillary walls.

## 5 Experimental Section

##### Capillary Hydrophobic Coating

The hydrophobic coating solution consisted of *n*‐octadecyltrichlorosilane (0.35 mL, 8.87 × 10^−4^ mol), which had been passed through a 0.22 µm syringe filter, dissolved in toluene (10 mL). To the solution, hydrochloric acid (HCl) (0.25 mL, 37% v/v, ≈12 m) was added, affording droplets of acid within the toluene solution. Before coating, the internal lumen of the capillary was flushed through with acetone, 2‐propanol, deionised water, ethanol, deionised water and then toluene, in that order, before the *n*‐octadecyltrichlorosilane coating solution was pumped through the capillary for 5 h, avoiding the uptake of the droplets of hydrochloric acid at the bottom of the solution vial. After 5 h, the capillary tube was flushed through sequentially with toluene, ethanol, ethanol‐deionised water (1:1), deionised water, and finally air. By applying this procedure to a planar clean glass substrate, water contact angles of >115° were measured, thus a hydrophobic coating was inferred to have formed on the internal lumen surface of the capillary when this procedure was used.

##### Two‐Photon Polymerization (2PP)

Nanoscribe technology was used to print both capillary valves of the nanopipette and microfluidic structure. IP‐Dip was placed on ITO‐coated glass and both structures were printed at 50% laser power using x25 objective. The structures were developed 10 min in propylene glycol monomethyl ether acetate (PGMEA) then placed for 2 h in a fresh PGMEA bath for further development. They were then rinsed in 2‐propanol for 2 min and then dried in a stream of air.

##### Statistical Analysis

The statistical calculations (mean and standard deviation) for the advancing and receding contact angles as well as the droplet volume change were performed using Microsoft Excel. The advancing and receding contact angle measurement data are expressed as the (*mean* ± *propagated error*) and the difference between the two groups was compared with Welch's *t*‐test.

## Author Contributions

A.B. proposed and designed the microfluidic device, performed the characterization, modeling, 2PP printing, assembly and writing. A.B. also assisted during the hydrophobic coating process. D.J.W. realized the hydrophobic coating of capillary and planar substrates, performed the contact angle measurements and supported A.B. with analysis and writing. E.Y. supervised all steps of the project and assisted with analysis. G.‐Z.Y. proposed and designed the fiberbot concept, initiated the project (microrobotics for surgery), secured funding, and supervised all steps of the project.

## Conflict of Interest

The authors declare no conflict of interest.

## Supporting information

Supporting Information

## Data Availability

All data needed to evaluate the conclusions in the paper are present in the paper and/or the Supporting Information. Additional data related to this paper may be requested from the authors.
