# Supplementary material for: Microfluidics at Fiber Tip for Nanoliter Delivery and Sampling
Source: Adv Sci (Weinh). 2021 Mar 15;8(10):2004643. doi: 10.1002/advs.202004643 (PMC8132067; doi:10.1002/advs.202004643)
Supplement: Supplementary file 1 — Supporting Information [file ADVS-8-2004643-s001.pdf]

© 2021 Wiley-VCH GmbH

ADVANCED SCIENCE
Open Access

Supporting Information

for *Adv. Sci.,* DOI: 10.1002/advs.202004643

Microfluidics at Fiber Tip for Nanoliter Delivery and Sampling

Antoine Barbot, Dominic Wales, Eric Yeatman, and Guang-Zhong Yang**

Supplementary Material

S1 Hydrophobic treatment, characterisation on flat surface

The contact angles in both the advancing and receding regimes were measured using a custom optical goniometer setup. The setup consisted of a Keyence VHX-2000 digital microscope that was used to record a video of the drops in the advancing and receding contact angle regimes at 28 frames a second. The setup was mounted on a sturdy bench within a cleanroom to minimise vibration and contamination. An initial drop of 10 μL was formed on the surface of the sample in a flat orientation, and then the volume of the drop was steadily increased and decreased three times each, whilst recording the video. It was ensured that the drop was in the advancing or receding state constantly. Such a method is similar to the method reported by Koch *et al.*[1] Taking into account the recommendations in the work of Korhonen *et al.*,[2] three frames from the video that showed instances of advancing contact angle, and three frames from the video that showed instances of receding contact angle were carefully selected, see Figure S1 below. The advancing and receding contact angles were then measured three times for each drop (N = 9 for both advancing and receding contact angles) using the angle measuring capabilities of Image J.[3] Overall average advancing and receding contact angles with the corresponded propagated errors were then calculated. It is noted that we propose that these propagated errors take into account measurement errors due to resolution of the images and pixel fitting errors, as highlighted in the work of Vuckovac *et al.*.[4]

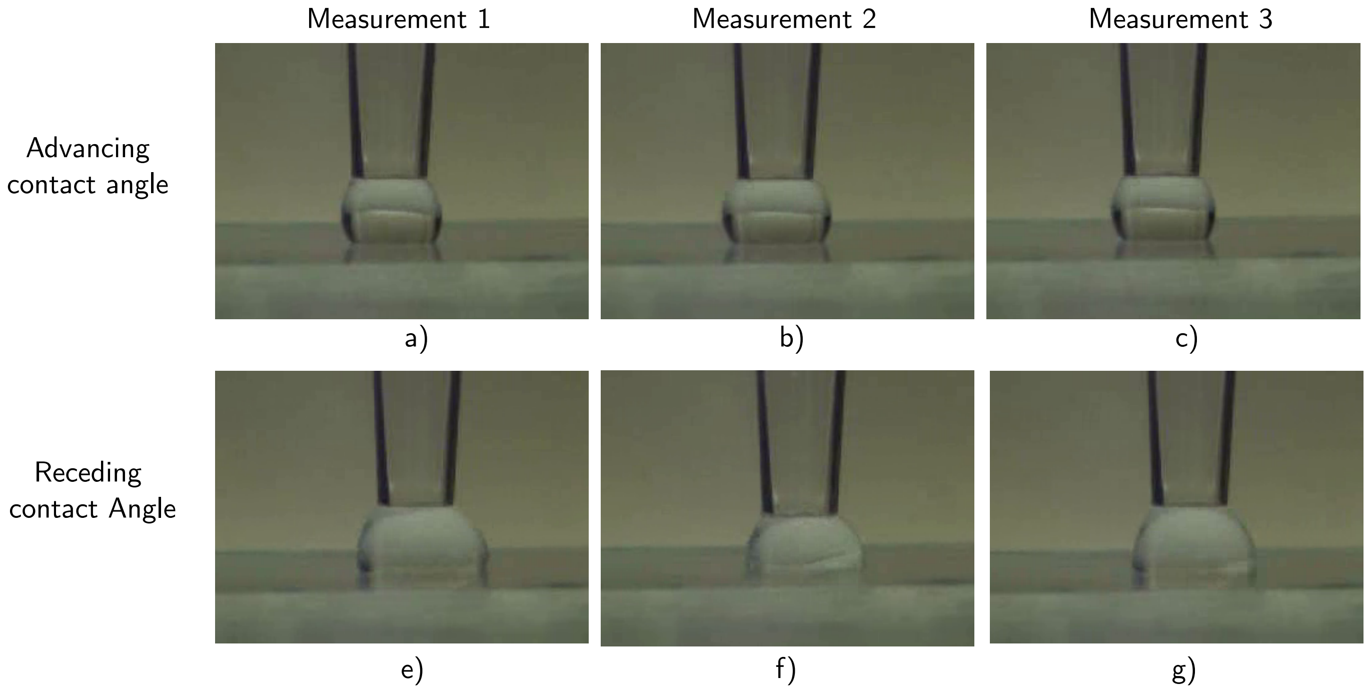

Figure S1: Frames from the video used for the measurement of the advancing and receding contact angle on a flat surface coated with the same hydrophobic coating as used for the capillaries. a-b-c) correspond to the three frames from which advancing contact angles were measured thrice (N = 9) whereas d-e-f) are the three frames from which receding contact angles were measured thrice (N = 9). The contact angle hysteresis is $(22.8 \pm 4.0)^\circ$.

S2 Second droplet impact on the speed

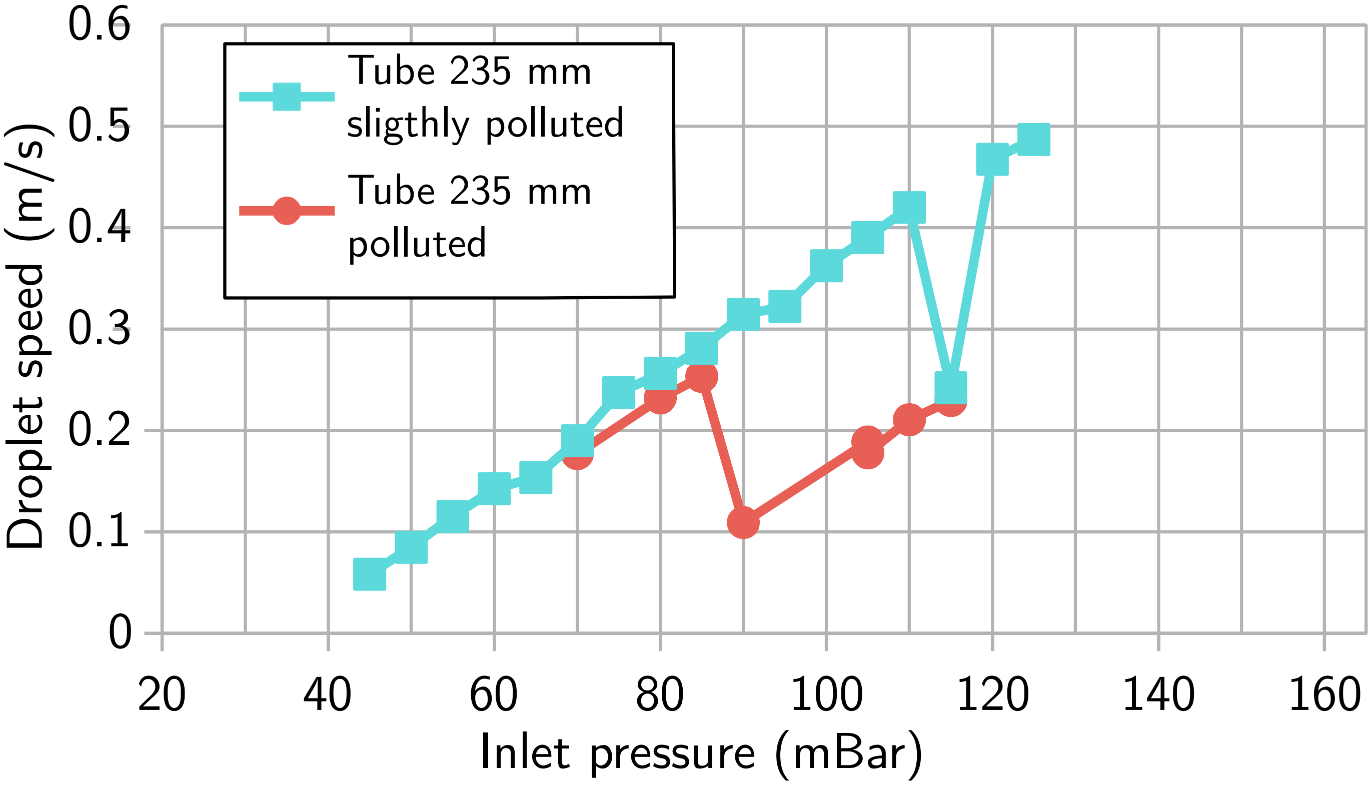

Figure S2: Speed in function of the driving pressure in polluted tube. The pollution induced a failure of the hydrophobic coating near the nanopipette passive valve resulting in the production of a 2nd droplet for higher pressure.

Figure S2 shows recorded droplet speed for capillaries polluted by the use of ink. A d is noticeable for higher pressure, especially in the more polluted capillary. Experimental record showed that this speed droplet corresponds to the presence of a smaller second droplet after the main one.

No major pollution was observable on the hydrophobic capillary, but the 2PP structure, and in particular the passive valve of the nanopipette, were greatly affected by the pollution. Indeed, their surroundings remained wet and were dried by the gas flow. Therefore, all the non-volatile particles accumulated and changed the local surface property.

This resulted in a defect on the hydrophobic property around the nanopipette which lead to an increase of the remaining water on the surface after the droplet ejection. This water triggered an instability of the wet surface when the gas passed in the middle of the capillary thus creating a second droplet.

This second droplet had a direct impact on the overall speed as the drag force from the contact line hysteresis was double. This can be clearly see in the Figure S2 where a second lower linear trend emerged in the polluted capillary with the same slope. This is to be expected as higher pressure induce quicker gas flow which is more likely to trigger the instability leading to a second droplet creation [5] In any case, no second droplet was present for driving pressure values below 90 mbar. Therefore we limited to this pressure for the characterisation of the microfluidic structure.

S3 Passive valve design

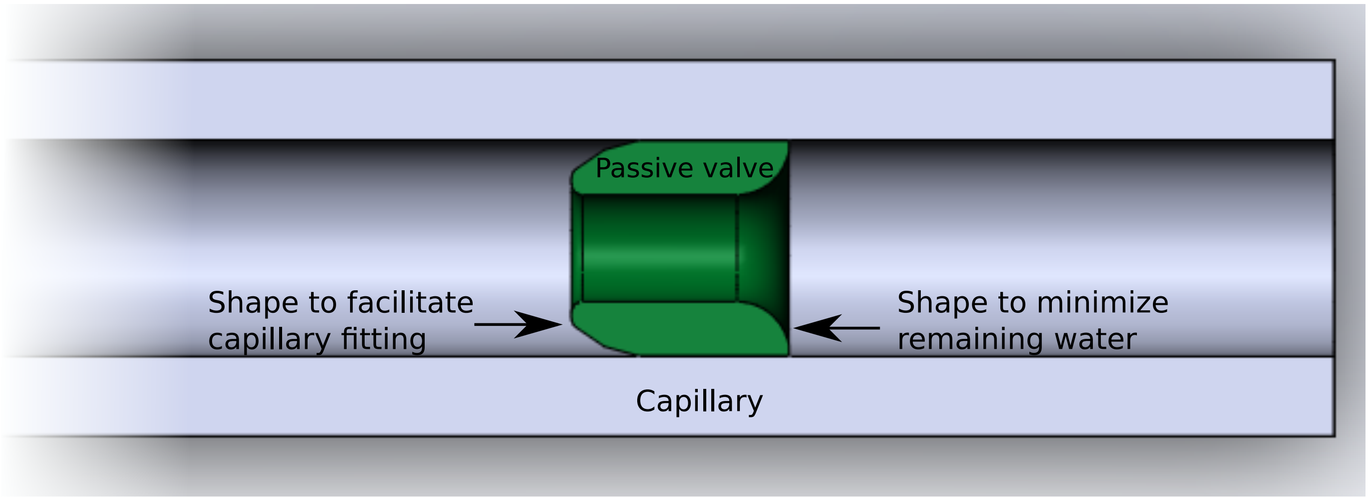

Figure S3: CAD cut view of the passive valve insert in the capillary to form the nanopipette. The shape of the valve was designed to allow both easy fitting into the capillary and small remaining wet volume after the ejection of the droplet

S4 2PP microfluidic structure assembly on capillary bundle

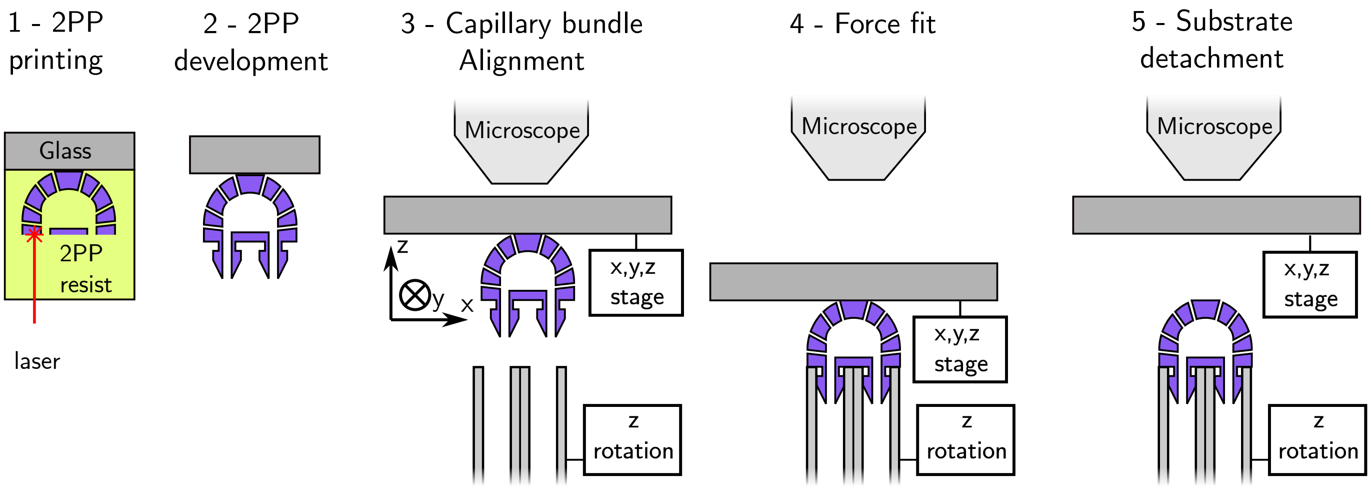

a)

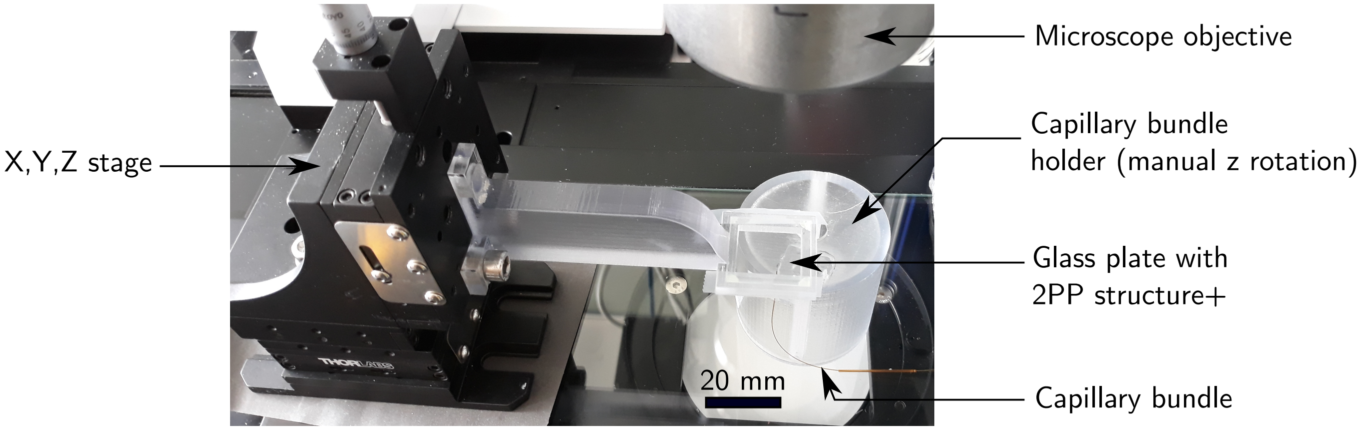

b)

Figure S4: Assembly of the 2PP microfluidic structure on the capillary bundle. a) shows schematic of the fabrication and assembly step. b) is a picture of the assembly configuration to align and force fit the 2PP microfluidic structure at the capillary bundle tip.

Using the reference on the figure S4 a) The fabrication steps are the following :

1. 2PP printing. Using Nanoscribe machine, the laser polymerised the resist to form the microfluidic structure with a micron level precision.
2. 2PP Development. A solvent was used to remove the non-polymerised resist.
3. Capillary bundle alignment. The capillary bundle was fixed on a holder that could be manually rotated to align its orientation with the microfluidic structure. The microfluidic structure substrate glass was fixed to a x,y,z manual micro-stage allowing for the alignment of the structure with the bundle with a micron resolution. A microscope was used to control these alignments. This stage corresponds to the view in the figure S4 b)
4. The Z part of the substrate stage was lower to force fit the bundle into the microfluidic structure.
5. The substrate stage was brought up leading to the detachment of the microfluidic structure from the glass substrate and its final assembly to the capillary bundle. Note that at this stage lateral

force apply by a needle could be used in case the adhesion between the microfluidic force and the glass was too strong.

S5 Delivery and sampling probe picture

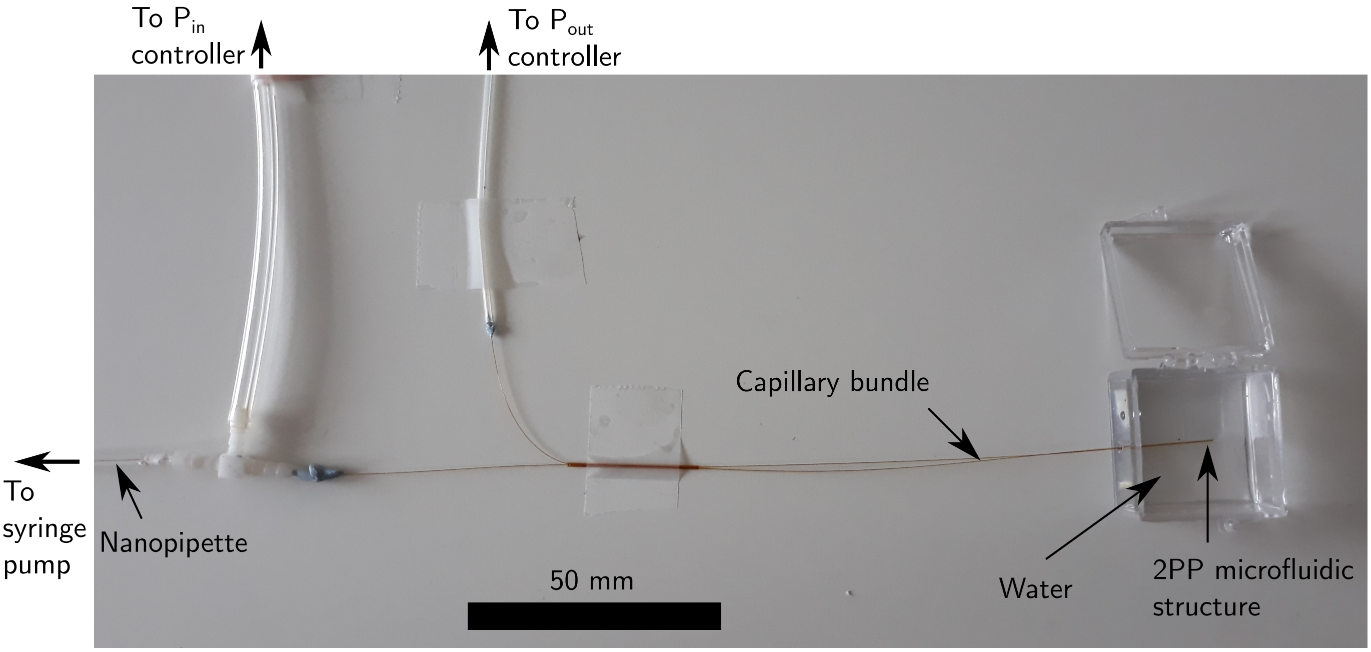

Figure S5: Picture of the full setup including the nanopipette, the capillary bundle and its tips with the 2PP microfluidic structure (too small to be visible). Connection to the pressure controller P_{in} and P_{out} are also highlighted

S6 Levitating droplet models and discussion

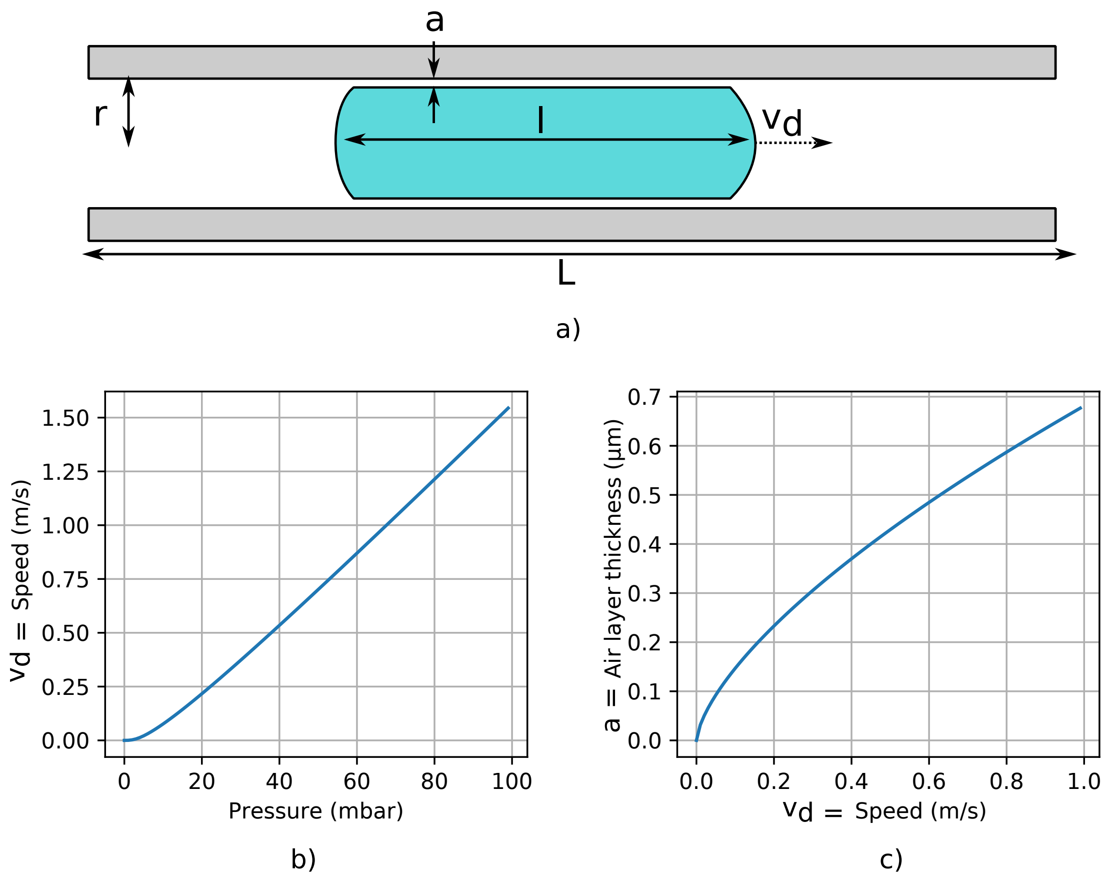

Figure S6: Levitating drops model. a) introduce the levitating droplet parameters where an air layer of thickness "a" prevent the adhesion between the droplet and the side of the capillary. b) Theoretical levitating droplet speed assuming the experimental parameters use for drop-speed tests. c) Theoretical layer thickness in function of the droplet speed

Above a critical capillary number (Ca), a small gas layer appears between a liquid droplet and the surface. This has been reported in hydrophobic microfluidic for a relatively low Ca number (inferior to 0.02) [6] and for larger Ca (0.3 to 4) in hydrophilic tubes [7]. Note that the Ca evolves linearly with the droplet speed, which corresponds in our experimental setup to 0.012 for 0.1 m/s droplet speed.

In the following we propose to derive the levitating droplet speed with the parameters of experimental setup with a capillary of length L and a driving pressure ΔP using the layer thickness calculation proposed by Favreau et al. [7] :

$$a = 0.643r(12C_a^g)^{2/3}, \tag{13}$$

with a being the gas layer thickness, r the capillary radius, $C_a^g = \frac{\mu_{air}V_d}{\sigma_{water}}$ the capillary number of the gas layer with the liquid interface.

In this model the liquid droplet speed V_d is uniform, therefore the only drag comes from the friction with the gas layer. The boundary condition of this gas layer imposes a null speed condition on the capillary surface and V_d on the droplet. Thus assuming a Couette flow in the layer the fluid drag on the droplet is :

$$\frac{-\mu_{gas}V_d 2\pi l}{a}, \tag{14}$$

with l being the length of the droplet.

By applying a force balance on the droplet, taking count of the pressure drop in the tube, we obtain the following:

$$\Delta P \pi r^2 - 8\mu_{gas} V_d L - \frac{\mu_{gas} V_d 2\pi l}{a} = 0, \tag{15}$$

with L being the overall length of the tube.

By using Equation 13 this equation gives the following equation for the speed drop :

$$V_d \frac{8\mu_{gas} L}{\pi r^2} + V_d^{1/3} 2l \frac{\mu_{gas}^{1/3} \sigma_{water}^{2/3}}{3.37 r^2} = \Delta P \tag{16}$$

By solving this equation we obtain the droplet speed evolution with the driving pressure which is drawn in Figure S6 b).

This model gives speed faster the experimental one of roughly an order of magnitude. This is probably explained as the air layer remains unstable and may break in several places due to the relative long droplet present in our experiment. Indeed, in the work of Favreau et al. [7] stable configurations seems to exist for droplets only few times longer than the capillary diameter, whereas in our work the droplet length is more than 10 times longer than the diameters. Moreover, Equation 13 gives the evolution of the droplet layer with the speed. In can be noted that this gas layer length is smaller than 1 μm which make it probably easier to break due to irregularity (either physical or chemical) of the surface.

In conclusion, we believe that our droplet maybe in a partial levitating condition with a region where the contact between the droplet and the capillary happen. This is probably due to the relatively long size of our droplet and surface irregularity. Further experiments with smaller droplet droplets examined with a high-speed camera should be performed to resolve this question. Controlling well the levitation of such droplets in a tube could help to improve the flow rate and delivery speed of such devices.

References for supplementary material

[1] B. M. L. Koch, A. Amirfazli, J. A. W. Elliott, *The Journal of Physical Chemistry C* **2014**, *118*, 32 18554.

[2] J. T. Korhonen, T. Huhtamäki, O. Ikkala, R. H. Ras, *Langmuir* **2013**, *29*, 12 3858.

[3] J. Schindelin, I. Arganda-Carreras, E. Frise, V. Kaynig, M. Longair, T. Pietzsch, S. Preibisch, C. Rueden, S. Saalfeld, B. Schmid, J.-Y. Tinevez, D. J. White, V. Hartenstein, K. Eliceiri, P. Tomancak, A. Cardonna, *Nature Methods* **2012**, *9* 676.

[4] M. Vuckovac, M. Latikka, K. Liu, T. Huhtamäki, R. H. A. Ras, *Soft Matter* **2019**, *15* 7089.

[5] J. G. Hagedorn, N. S. Martys, J. F. Douglas, *Phys. Rev. E - Stat. Physics, Plasmas, Fluids, Relat. Interdiscip. Top.* **2004**, *69*, 5 18.

[6] P. Tirandazi, C. H. Hidrovo, *Journal of Micromechanics and Microengineering* **2017**, *27*, 7 075020.

[7] P. Favreau, A. Duchesne, F. Zoueshtiagh, M. Baudoin, *arXiv preprint arXiv:2002.12839* **2020**.
